# Mechanical Properties of PolyJet 3D-Printed Composites Inspired by Space-Filling Peano Curves

**DOI:** 10.3390/polym13203516

**Published:** 2021-10-13

**Authors:** Changlang Wu, Truong Tho Do, Phuong Tran

**Affiliations:** 1 School of Civil and Infrastructure Engineering, RMIT University, Melbourne, VIC 3000, Australia; s3819965@student.rmit.edu.au; 2 College of Engineering and Computer Science, VinUniversity, Hanoi 14000, Vietnam; truong.dt@vinuni.edu.vn; 3 Advanced Manufacturing Precinct, School of Engineering, City Campus, RMIT University, Melbourne, VIC 3000, Australia; 4 Centre for Innovative Structures and Materials, School of Engineering, RMIT University, Melbourne, VIC 3001, Australia

**Keywords:** Peano curve, composite, PolyJet 3D printing, rule of mixture, multi-material printing, additive manufacturing

## Abstract

This paper proposes a design of novel composite materials inspired by the Peano curve and manufactured using PolyJet 3D printing technology with Agilus30 (flexible phase) and VeroMagentaV (rigid phase) materials. Mechanical properties were evaluated through tensile and compression tests. The general rule of mixture (ROM) for composites was employed to approximate the tensile properties of the hybrid materials and compare them to the experimental results. The effect of reinforcement alignments and different hierarchies are discussed. The results indicated that the 5% inclusion of the Peano reinforcement in tensile samples contributed to the improvement in the elastic modulus by up to 6 MPa, but provided no obvious enhancement in ultimate tensile strength. Additionally, compressive strengths between 2 MPa and 6 MPa were observed for compression cubes with first-order reinforcement, while lower values around 2 MPa were found for samples with second-order reinforcement. That is to say, the first-order reinforcement has been demonstrated more effectively than the second-order reinforcement, given the same reinforcement volume fraction of 10% in compression cubes. Different second-order designs exhibited slightly different mechanical properties based on the ratio of reinforcement parallel to the loading direction.

## 1. Introduction 

Fractal patterns exist everywhere in nature in various ways, such as in spider webs, the Milky Way galaxy, and coastlines. The concept of fractal was first introduced by Mandelbrot [1] in 1977. He defines it in the book *Fractals in Physics* as [2]:

‘Fractal is a structure comprised of parts that, in some manner, are similar to the whole of this structure.’ (p. 250)

Self-similarity, the main attribute of fractal patterns, indicates that the geometry consists of a unit structure repeating itself in different scales [3]. The self-similarity feature can be found in many objects, such as Russian matryoshka dolls, the Koch snowflake, etc. However, fractal structures were not applied to industries until some theoretical analyses and experiments were conducted recently [4]. Space-filling curves are special cases of fractal structures, which are characterized by a unique property that, after an infinite number of iterations, a finite area would be filled with a curve of infinite length. The most famous space-filling curves include the Peano curve, the Hilbert curve, and the Moore curve.

**Figure 1:**
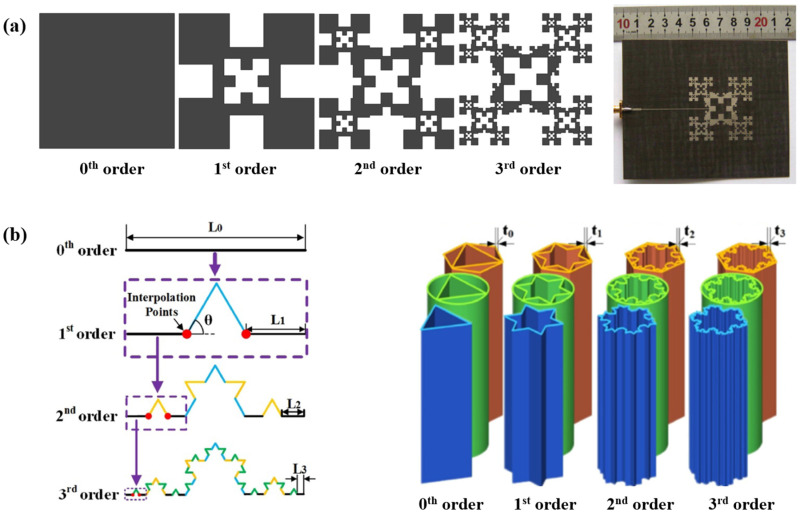
(**a**) Koch and Sierpinski patterns inspired antenna design [9], reproduced courtesy of The Electromagnetics Academy; (**b**) Koch snowflake inspired thin-walled structure design for energy absorption [51].

In the past two decades, scientists have embraced the study of fractal geometries, with respect to electronics design (Figure 1a). Studies reveal that fractal-shaped antennas show superior properties from their geometrical attributes. The self-similarity characteristic of fractal patterns contributes to a multiband feature of the corresponding antennas [5,6,7,8], while the high convoluted shape and space-filling properties of certain fractal curves allow for the reduction of the miniaturization of microstrip antennas, resonators, and filters [9,10,11,12]. These properties show great potential for designing multiband antennas, frequency-selective surfaces, and reducing the size of antennas. Since fractal geometry was first introduced to antenna array design by Kim and Jaggard [13], various space-filling curve designs have been utilised to improve the performance of antennas, including the Peano curve [14,15,16,17,18], Hilbert curves [14,15,16,19], the Koch curve [8,20,21], the Gosper curve [22,23], the Moore curve [10,16], the Sierpinski curve [6,7], the Minkowski curve [24,25], the Greek cross [16], and combinations of multiple geometries, such as the Peano-Gosper curve [26,27,28], the Koch-Sierpinski shape [9,29,30], and the Hilbert-Minkowski pattern [31]. Moreover, the mechanical stretchability of space-filling shaped electronics has attracted growing interest from researchers to achieve both advanced electronic function and compliant mechanics. Fan et al. [16] demonstrated that fractal-based structures bonded to pre-strained elastomers enable higher levels of elastic deformation. It was also indicated that fractal-based layout could provide a strategy to integrate hard and soft materials. Similar studies were conducted to investigate the stretchability of fractal-based stretchable electronics [32,33,34,35]. 

In addition to its value in electronics, fractal-based geometry has also been adapted for novel material design in recent studies. Fractal patterns appear in many natural materials, such as shells and bones. These natural materials have attracted considerable attention from scientists due to their excellent mechanical properties. Huiskes et al. [36] claimed that the fractal morphology of trabecular bone contributed partly to its mechanical efficiency. Following this theory, Farr [37,38,39] applied fractal principles to structure designs, showing the improvement in mechanical efficiency under gentle compressive loading conditions. So far, many studies have been conducted on fractal-like hierarchical honeycombs regarding both in-plane and out-of-plane properties [40,41,42,43,44,45,46,47,48,49]. In 2015, Meza et al. [50] created structural metamaterials with exceptional strength, stiffness, and damage tolerance from materials in which unit cells were organized into a self-repeating geometry. Wang et al. [51] proposed a Koch-curve hybrid structure as shown in Figure 1b, indicating its energy absorption capability and lightweight feature. Additionally, fractal-like patterns have also been demonstrated to be promising in the design of stronger interlockings. Typical examples are the hierarchical suture joints inspired by ammonite fossils [52] and the 3D-printed Koch curve interlockings [53]. It was shown that the load-bearing capacity of the interlocking could be effectively increased via fractal design. Recently, the well-known 3D fractal structures, which are called Menger Sponge cubes, were 3D printed using direct laser lithography [54] and demonstrated superior energy absorption ability.

The emergence of additive manufacturing realizes the fabrication of structures with complex geometries and exceptional engineering properties, which could not be achieved by conventional manufacturing methods. Recent studies regarding multi-material 3D printing have demonstrated its superior function in creating structures/materials with tunable mechanical properties [55]. For example, multi-material fused deposition modelling (mFDM) 3D printing technology was utilised by Zhang et al. [56] to manufacture functionally gradient composites with user-defined mechanical properties. More studies have been conducted using material-jetting technology. In 2019, Skylar-Scott et al. [57] proposed an inkjet multi-material, multi-nozzle 3D printing method to generate origami structures, using two different viscoelastic epoxy inks for flexible hinges and rigid faces, respectively. The resulting structures showed the capability during compression in terms of large deformation in the hinges and multiple folding cycles before failure. Later, Yuan et al. [58] used PolyJet technology to fabricate composites with two photopolymers, VeroBlack and TangoPlus. According to their study, programmed shape-memory behaviours were achieved by the 3D printed structures.

Previous studies have successfully demonstrated the potential of fractal patterns in material design, whereas a limited variety of self-similar shapes have been explored. Despite the fact that multi-material printing exhibits the capability to create structures/materials with tunable properties, most studies focused on single material design and fabrication. In this study, we propose a novel design of 3D-printed composites. The hybrid materials feature a space-filling curve modified reinforcement and are manufactured using PolyJet 3D printing technology. Experiments, microscopy, and analytical models are conducted to investigate the mechanical properties of innovative materials. The results of this study provide insight into a continuous-curve-reinforced polymer composite, which has potential application in biomedical [59], automotive [60], and aerospace engineering [61].

## 2. Methods

### 2.1. Material Design and Fabrication

**Figure 2:**
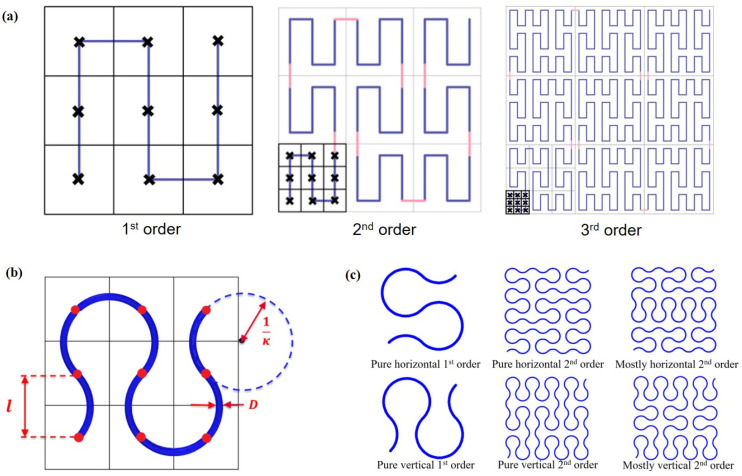
(**a**) Construction of the first three orders of Peano curves; (**b**) schematic design of a smoothed Peano curve, including control points (red dots), the side length of a small square (*l*), arc curvature (*k*), and diameter (*D*); (**c**) variants of Peano curves at different orders to be investigated in this study.

The Peano curve, which was introduced by an Italian mathematician Giuseppe Peano, was the first space-filling curve to be discovered. The set of curves consists of many orders, which can be constructed following a sequence of steps as shown in Figure 2a. Considering mechanical properties and manufacturing issues, all the sharp edges in the original Peano curves are smoothed using arcs as shown in Figure 2b. Rhino with the Grasshopper plugin is employed as the CAD software. Figure 2b defines three geometric parameters, i.e., the side length of a small square (*l*), arc curvature (*k*), and diameter (*D*). Six patterns of Peano curves are to be investigated in this study with respect to various orientations and different hierarchies as shown in Figure 2c.

The proposed first order and second order Peano curves are designed to act as a hard reinforcement, which is embedded in a soft-material matrix in order to investigate the mechanical performance of 3D-printed composites. Stratasys J750 Digital Anatomy 3D printer, provided by Stratasys Ltd., Rehovot, Israel, is a PolyJet 3D printer and was used to fabricate all the samples. This printer has four inkjet heads and two UV light sources, allowing multi-material 3D printing from a wide range of available materials. J750 is also capable of generating complex geometries with microscopic layer resolution, down to 0.014 mm. All the samples were manufactured with two different materials, VeroMagentaV (VMV) and Agilus30 (A30). VMV is a rigid and opaque photopolymer, while A30 is a rubber-like polymer. VMV is from the family of Vero; available in seven hues, including blue, white, black, grey, cyan, magenta, and yellow, the Vero family shares similar mechanical, thermal, and electrical properties. Here, VeroMagentaV is selected to offer a more saturated and vibrant colour compared to the transparent A30.

**Figure 3:**
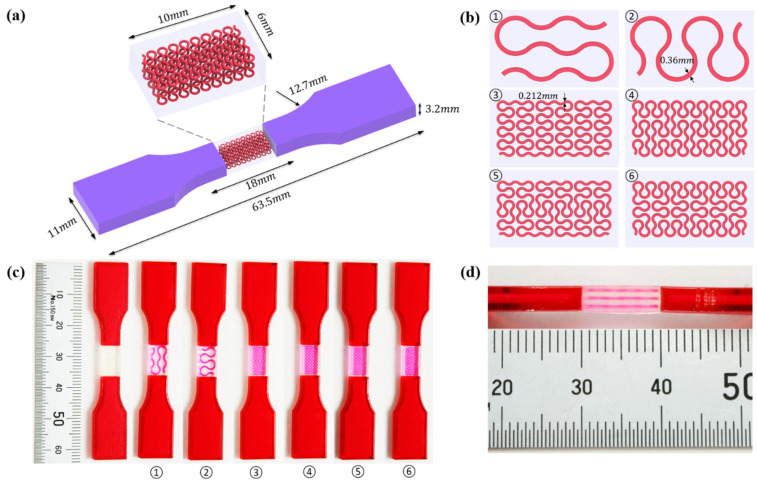

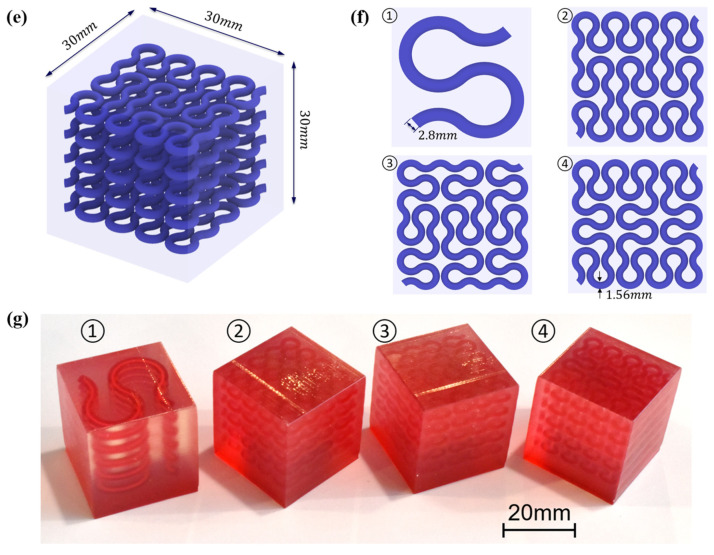
(**a**) Dimensions of a tensile specimen; (**b**) plan views of six different hybrid structures using different Peano patterns; (**c**) 3D-printed tensile samples, including pure A30 and composites with VMV reinforcement embedded inside A30; (**d**) side view of the gauge section for the hybrid materials, showing the three-layer reinforcement; (**e**) dimensions of the compression cube; (**f**) plan views of four different hybrid structures showing the first order reinforcement with a reinforcement diameter of 2.8 mm and 1.156 mm for all three second order designs; (**g**) pictures of 3D printed samples (from left to right, corresponds to case 1 to case 4, respectively).

So far, no standard of the tensile test has been established for 3D-printed multi-material structures/materials. In this study, tensile samples are designed according to the ASTM D638, with variations from the literature [62,63] as shown in Figure 3a. Two categories of samples are prepared for further analysis. First, homogeneous A30 samples are printed to capture their individual mechanical properties, thereby providing a reference to composite materials. Then, six designs of hybrid samples (Figure 3b) are fabricated, with A30 serving as the matrix of gauge section, and VMV as both the reinforcements and extended sections. Figure 3b schematically shows the gauge sections of six heterogenous designs, reinforced with differently orientated and hierarchical Peano curves, including pure vertical first order, pure horizontal first order, pure vertical second order, pure horizontal second order, mostly vertical second order, and mostly horizontal second order. The reinforcements are distributed in three layers at a spacing of 1 mm. All the hybrid tensile structures were reinforced with VMV at a volume fraction of 5%. Thus, the diameters for the first and second order Peano reinforcements are 0.36 mm and 0.212 mm, respectively. Figure 3c depicts the 3D-printed tensile samples.

The compression specimens are designed as cubes with a side length of 30 mm, as shown in Figure 3e. The cubic matrix is A30, which is reinforced by five-layer VMV Peano curves at a spacing of 5 mm. Four different infills, with a volume fraction of 10%, are introduced as shown in Figure 3f,g. The first order Peano reinforcement has a diameter of 2.8 mm, while the second order has a diameter of 1.56 mm. 

### 2.2. Mechanical Testings

In order to investigate the mechanical properties of Peano reinforced hybrid materials, tensile and compression tests were conducted using the Universal Instron testing machine. Tensile tests were controlled with a displacement rate of 1 mm/min until a failure happens, while the uniaxial compression tests were performed with a rate of 1.3 mm/min until strain reaches 60%. Compression tests were performed from three axial directions (Table 1) considering the anisotropic property of the cubic designs. Fives samples for each type of design were tested to minimise the experimental artifacts.

### 2.3. Rule of Mixture for Composites

In order to provide theoretical references for experimental results, the Rule of Mixture (ROM) was adapted in this study to approximate the elastic properties of composite materials. Based on different assumptions, both the upper and lower bounds of the elastic modulus for composites could be found. When the load is applied longitudinally to the fibre, the ROM defines the highest elastic modulus of the composite according to the iso-strain assumption:

(1) $$E_{c,\;\;max}=fE_{f}+\left(1-f\right)E_{m},$$

where $E_{c,min}$ denotes the upper bound of the elastic modulus of the composite; $f=\frac{V_{f}}{V_{f}+V_{m}}$ is the volume fraction of reinforcement; $E_{f}$ is the elastic modulus of the VMV reinforcement; and $E_{m}$ is the elastic modulus of the A30 matrix. It should be noted that Equation (1) can also be applied to predict other elastic properties, for example, the ultimate tensile strength.

When the load is applied transverse to the fibre, the lower bound of the elastic modulus could be estimated using the following equation according to the iso-stress assumption:

(2) $$E_{c,min}={\left(\frac{f}{E_{f}}+\frac{1-f}{E_{m}}\right)}^{-1},$$

where $E_{c,min}$ denotes the lower bound of the elastic modulus of the composite.

In this study, the theoretical range of the elastic moduli of the novel hybrid materials is predicted by Equation (1) and Equation (2). The experimental results are expected to sit in between the range. Additionally, the ultimate tensile strength of the composite materials is estimated using the ROM by Equation (1). In the next section, the approximations from the analytical models and experiments are compared with detailed discussions on the discrepancy.

## 3. Results and Discussion

### 3.1. Tensile Test Results and Discussion

Herein, stress-strain curves obtained in tensile tests are presented and compared. Five specimens for each design were tested and the results are illustrated with details of the average stress and standard deviation. The elastic moduli and ultimate tensile strengths are captured from the experiments and then compared to theoretical estimations. Moreover, crack propagations and fracture surfaces are investigated with representative microscope images provided.

**Figure 4:**
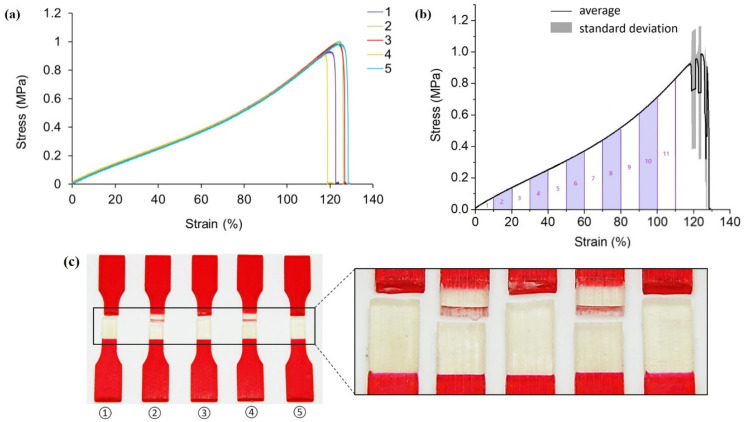
(**a**) Stress-strain curves obtained from tensile testing tests on non-reinforced A30 samples; (**b**) average tensile stress—strain curve and standard deviation of all five testings; (**c**) pictures of failed tensile samples with zoom-in at the locations of fracture.

Figure 4 shows the tensile testing results for pure A30 samples. Despite the slightly different elongations of the five specimens, the non-linear responses of all five tests are repeatable. As revealed by Figure 4a, specimen two experiences the maximum stress of 0.94 MPa, while specimen four experiences the least at 0.85 MPa.

Since A30 is a rubber-like, hyper-elastic material, it is typically not described using Young’s modulus and Poisson’s ratio [64]. To be more specific, the elastic modulus of A30 is not constant but changes with strain. In order to approximate the value, the average stress-strain curve before fracture is divided into eleven segments. Each segment corresponds to a 10% strain change as shown and numbered in Figure 4b. The stress-strain curve within each segment is assumed to be linear so that the elastic moduli of A30 could be estimated. Results from the eleven segments approximate a range from 0.56 MPa to 1.18 MPa for the elastic modulus of A30.

Figure 4c depicts the failure samples with a magnified picture at the gauge sections. Fractures are identified to happen at different locations, including the gauge section (specimen two and four from left to right), close to the extension part (specimen one and three from left to right), and also at the interface of two different materials (specimen five). This phenomenon could ascribe to 3D printing defects.

**Figure 5:**
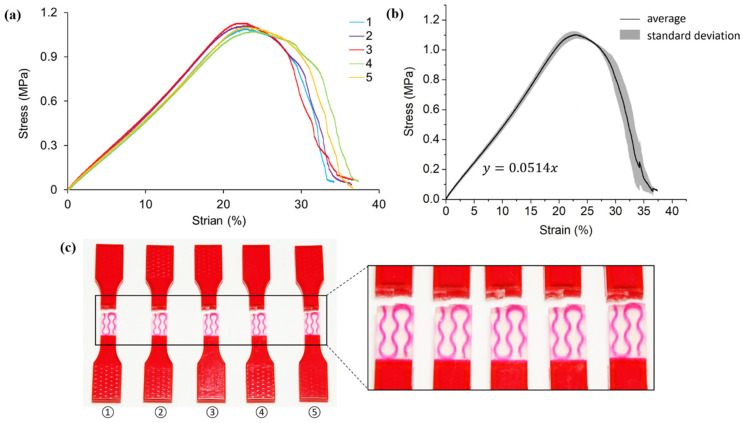
(**a**) Stress-strain curves obtained from tensile testing tests on hybrid case one (pure horizontal first order) samples; (**b**) average tensile stress-strain curve and standard deviation of all five testings; (**c**) pictures of failed tensile samples with zoom-in at the locations of fracture.

Figure 5 shows the tensile test results of hybrid case one samples, which introduce the first order pure horizontal Peano VMV reinforcement into the A30 matrix. The responses of all five specimens are similar, particularly the elastic deformation stage (strain less than 20%) as suggested by the stress-strain curves in Figure 5a. All specimens experience similar maximum tensile stress of 1 MPa, approximately.

An average elastic modulus of 5.14 MPa is captured in Figure 5b. Compared to the results of homogenous A30 samples, there are improvements in both the ultimate tensile stress and elastic modulus. As the results imply, the introduction of embedded VMV reinforcement in hybrid case one contributes to an enhancement in both the tensile strength and stiffness.

Different from homogenous A30 samples, all the fractures of case one samples are located in the A30 matrix and near the edge of the gauge section (Figure 5c). In other words, failure only happens between the edge of the reinforcement and the extension. This phenomenon could be explained by the non-effective stress transfer between A30 and VMV. According to the material datasheet provided by Stratasys Ltd., VMV has a much higher strength and stiffness than A30. As a result, crack would initiate in A30 instead of VMV after the elastic deformation phase. Given the fact that there is no reinforcement existing near the extensions, these cross-sections are the most vulnerable when subjected to tensile force. Therefore, the crack initiates and propagates in the matrix near the extension until it totally fails.

**Figure 6:**
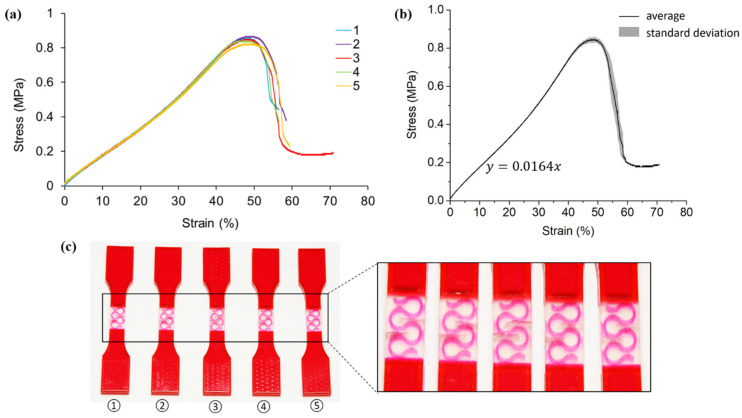
(**a**) Stress-strain curves obtained from tensile testing tests on hybrid case two (pure vertical first order) samples; (**b**) average tensile stress-strain curve and standard deviation of all five testings; (**c**) pictures of failed tensile samples with magnification at the locations of fracture.

Figure 6 describes the tensile test results on the hybrid case two samples, featuring the first order pure vertical Peano curve.

Similar to hybrid case one, the elastic responses of all five specimens are consistent (less than 40% strain) as indicated in Figure 6a. An average elastic modulus of 1.64 MPa is identified in Figure 6b. The elastic modulus of hybrid case two is increased by 0.96 MPa compared to pure A30, which is attributed to the introduction of reinforcement. However, hybrid case two is less stiff than case one. Given that both case one and case two have the same hierarchy and volume fraction of reinforcement, it could be inferred that the orientation of the Peano curves has a significant influence on the elastic modulus.

As the gaps between the curved reinforcement are bigger than those between the reinforcement and extensions, the A30 within the reinforcement gaps is more vulnerable. Therefore, fractures of the second case happen in A30 in between the curved reinforcements (Figure 6c) rather than near the extensions as in case one. With respect to maximum tensile stress, all specimens experience similar values of around 0.8 MPa. Unlike the hybrid case one design, the strength of hybrid case two is lower than pure A30 samples. In hybrid materials, crack initiates in A30 in between the reinforcement and propagates until getting close to the reinforcement. In homogenous A30 samples, crack keeps propagating until a fracture happens since there is no reinforcement at any cross-section. However, the VMV reinforcement along the loading direction in hybrid case 2 confines the deformation of A30 in the transverse direction. Therefore, the ultimate tensile strength decreases compared to the homogeneous A30 samples.

**Figure 7:**
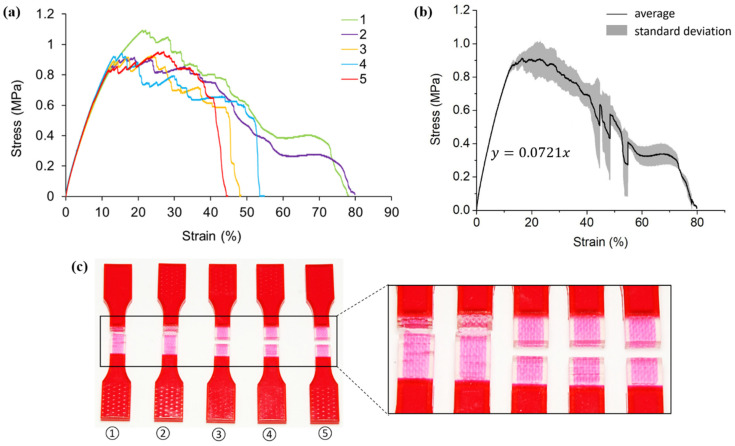
(**a**) Stress-strain curves obtained from tensile testing tests on hybrid case three (pure horizontal second order) samples; (**b**) average tensile stress-strain curve and standard deviation of all five testings; (**c**) pictures of failed tensile samples with zoom-in at the locations of fracture.

The tensile test results of hybrid case 3 samples are exhibited in Figure 7. The stress-strain curves (Figure 7a) reveal that all specimens experience the same stress roughly before the strain reaches 10%. The average elastic modulus is captured to be 7.21 MPa as shown in Figure 7b. Similar to hybrid case one and case two, the inclusion of VMV reinforcement in case three improves the structural stiffness of the coupon samples. As indicated by the comparison between cases one and three, the second order reinforcement contributes more to the stiffness than the first order reinforcement.

In the plastic deformation stage, the responses of different specimens are significantly different as described by the large standard deviation in Figure 7b. Specimen one experiences the largest maximum stress of 1.03MPa, while the others share an average around 0.88MPa. It seems that the inclusion of second-order pure horizontal reinforcement contributes little to the ultimate tensile strength compared to homogenous A30. Additionally, specimens one and two reach much greater elongations than the rest. It is worth noticing c that specimen one and two break near the extensions, while the other three fail closer to the middle of the gauge sections. Different fracture locations are mainly owed to the manufacturing defects.

Different from smooth stress-strain curves obtained for pure A30, hybrid case one, and case two, the strongly jagged pattern of stress-strain curves is observed for all case three specimens in the plastic deformation phase. Once the crack initiates in A30, it propagates perpendicular to the tensile force direction until it encounters the VMV reinforcement. Due to the arc design of the Peano curve, the straightening of the reinforcement is involved first and followed by material stretching. This process leads to a decrease in stress and a certain amount of increase afterward. Since the reinforcement design in case three is more complicated than that of case one and case two, cracks happen and develop at more cross-sections, round after round. Consequently, the stress-strain curves, after the crack initiation, are wavy until fractures happen.

**Figure 8:**
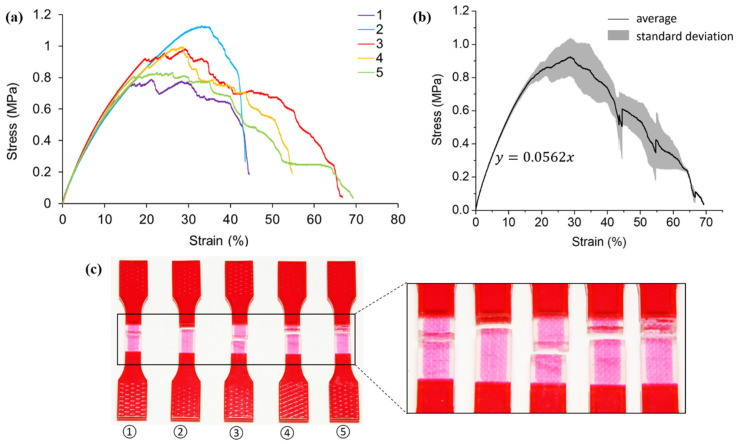
(**a**) Stress-strain curves obtained from tensile testing tests on hybrid case four (pure vertical second order) samples; (**b**) average tensile stress-strain curve and standard deviation of all five testings; (**c**) pictures of failed tensile samples with zoom-in at the locations of fracture.

Similarly, the stress-strain curves (Figure 8a) for the elastic stage are repeatable for hybrid case four samples. An average elastic modulus of 5.62 MPa is captured (Figure 8b). Comparing with the homogeneous A30 (0.56 ~1.18 MPa), the design of VMV reinforcement in case four significantly improves the structural stiffness. Again, the higher elastic modulus captured for case four than case two demonstrates that the second order reinforcement contributes more to the stiffness than the first order designs.

The five specimens experience different ultimate tensile stress, ranging from around 0.75 MPa to 1.1 MPa. Specimen two elongates the least (45%) with the maximum ultimate tensile stress. As observed from Figure 8c, specimen two fails instead of within the gauge section but nearly at the interface of the gauge section and extension. That is to say, the entire gauge section deforms elastically until crack happens at the A30 cross-section close to the extension. Moreover, the elastic deformation stage of specimen two ends at the strain of 30%, which is longer than the other four specimens. The plastic response of specimen two is dominant to A30, thereby resulting in a less jagged stress-strain curve compared to the others. Differently, cracks happen earlier and develop at the cross-section with VMV reinforcement for specimens one, three, four, and five. This explains the lower ultimate tensile stress and wavy stress-strain curves experienced by these four specimens. As the results demonstrate, the second order pure vertical reinforcement design contributes little to the ultimate tensile strength.

**Figure 9:**
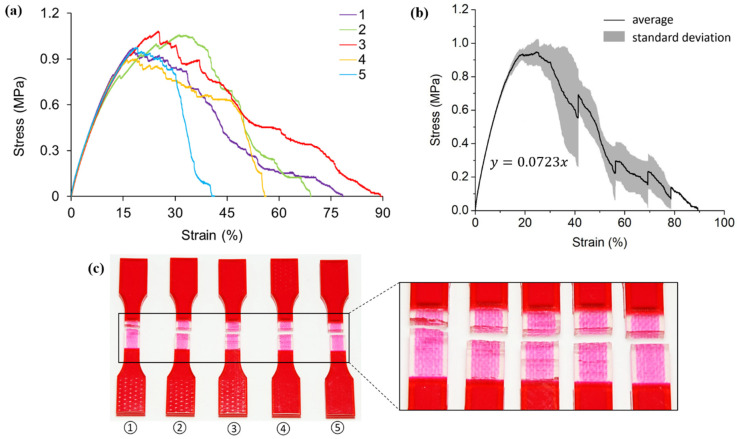
(**a**) Stress-strain curves obtained from tensile testing tests on hybrid case five (mostly horizontal second order) samples; (**b**) average tensile stress-strain curve and standard deviation of all five testings; (**c**) pictures of failed tensile samples with magnification at the locations of fracture.

Figure 9 depicts the tensile test results of the hybrid case five samples, which includes the second order mostly horizontal Peano VMV reinforcement into the A30 matrix. The repeatable response before a strain of 10% (Figure 9a) captures an average elastic modulus of 7.23 MPa (Figure 9b). Comparing to pure A30 (0.56~1.18 MPa), the design of VMV reinforcement in case five significantly improves the structural stiffness of the coupon samples.

However, the ultimate tensile stress experienced by all five specimens is not obviously increased in hybrid case five. As illustrated in Figure 9c, all specimens fail within the gauge section and at the cross-section with VMV reinforcement. Nevertheless, the elongation of different specimens varies widely between a strain of 40% to 90%. It could be observed from the magnified picture of specimen five that no obvious crack happens at other cross-sections except for the final failure. As a result, specimen five fractures at the smallest strain. On the other hand, specimen three experiences the greatest elongation of 90% with many cracks at different cross-sections.

**Figure 10:**
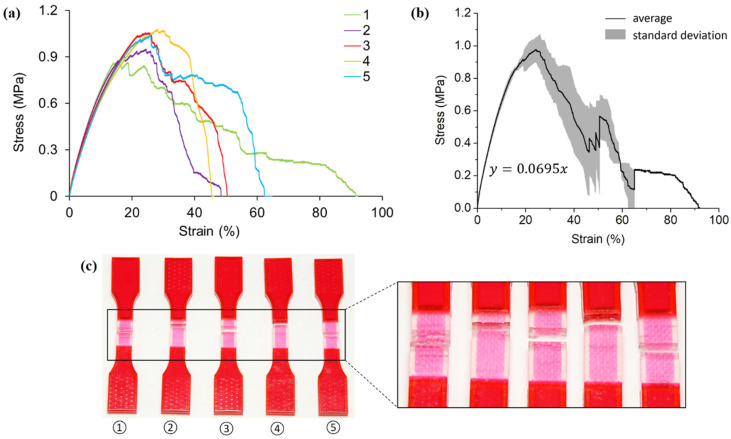
(**a**) Stress-strain curves obtained from tensile testing tests on hybrid case six (mostly vertical second order) samples; (**b**) average tensile stress-strain curve and standard deviation of all five testings; (**c**) pictures of failed tensile samples with zoom-in at the locations of fracture.

The tensile stress-strain curves and failed samples of the hybrid case six design are presented in Figure 10. The elastic stage, corresponding to a strain less than 10%, is quite repeatable for all specimens (Figure 10a) with an average elastic modulus of 6.95 MPa (Figure 10b). Obviously, the VMV reinforcement in case six enhances the stiffness of the coupon samples compared to homogenous A30. The elastic moduli of case four and case six are very close, owing to similar reinforcement alignments (orientation and hierarchy).

The five specimens experience different ultimate tensile stress as well as elongations. Specimen one reaches the highest fracture strain of more than 90%, while its ultimate stress is the smallest among all at 0.82 MPa. On the contrary, specimen four exhibits the highest strength at 1.01 MPa and goes through the least elongation. Furthermore, specimen four fails near the extension whereas others fail within the gauge section at the cross-section containing reinforcement (Figure 10c). The wavy patterns of all stress-strain curves could be explained by the crack propagation from A30 to VMV as mentioned before. Different fracture locations are likely to result from manufacturing defects within the gauge section.

**Figure 11:**
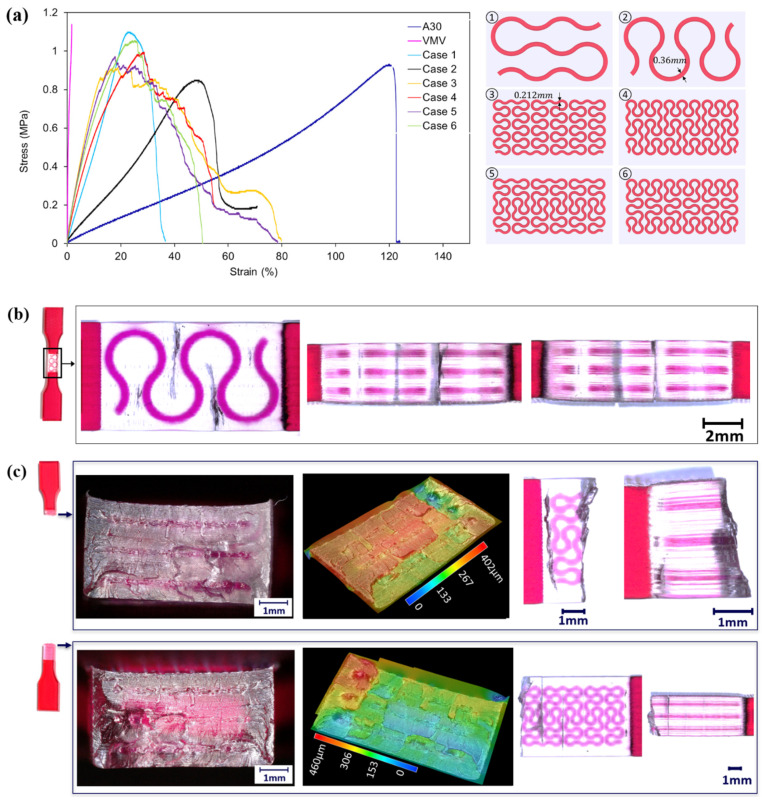
(**a**) Comparisons of tensile stress-strain curves of homogenous A30 (Agilus30), VMV (VeroMagentaV), and six hybrid designs (plan views of gauge sections for different hybrid cases are shown on the right); (**b**) microscopic images (50× magnification) taken from the top and side views of the case 2 specimen, showing the cracks happening in A30 stopping near VMV reinforcement; (**c**) microscope images (50× magnification) taken from fracture surfaces, top and side views of case 5 specimen, showing the uneven fracture surfaces, crack initiating points, and crack distributions.

Herein, Table 2 compares the final experimental results with the analytical predictions on elastic modulus and ultimate tensile strength of the hybrid materials. Figure 11a schematically summaries the responses of materials subjected to tensile loadings. Data regarding VMV is adopted from Tee et al. [62] to help better understand the mechanical properties of the novel hybrid materials.

As revealed by the table, elastic moduli obtained from experiments are within the analytical prediction ranges but much closer to the lower bounds. All the hybrid materials exhibit higher elastic moduli than homogeneous A30, indicating the positive effect of VMV reinforcement embedded in the A30 matrix. For the hybrid materials with the same hierarchical reinforcements, the higher ratio of the reinforcement parallel to the loading direction and perpendicular to the loading direction leads to a higher elastic modulus. However, it is not applicable to materials with different order reinforcements. Samples reinforced by the first order Peano curves (case one and case two) yield smaller elastic moduli than the second ones (case 3–6), even though the ratio for the former ones is higher than the latter. Results demonstrate that the second order reinforcement designs are more effective than the first order despite having the same volume fraction (5%). In addition, hybrid case three and case five exhibit the highest stiffness among all. It can be concluded that the second order pure horizontal and the second order mostly horizontal reinforcement are the most effective designs in terms of stiffness enhancement.

With regard to ultimate tensile strength (UTS), Table 2 reveals that there is no obvious improvement in hybrid materials compared to homogenous A30. Particularly, the reinforcements in case two and case four contribute negatively to UTS. This phenomenon is attributed to the reinforcement along the tensile force direction that confines the transverse deformation of A30 in the gauge section. Hybrid case one exhibits the highest tensile strength, whereas all specimens fail at the cross-section without any reinforcement. The results indicate that the coupon sample design of case one could not transfer the stress from the A30 matrix to VMV reinforcement effectively. Moreover, experimental results are lower than the theoretical estimations due to manufacturing defects in samples. Even though the improvement in tensile strength is not remarkable by the inclusion of reinforcement, a clear upward trend of UTS is identified with the increasing ratio of reinforcements parallel to and perpendicular to the loading direction.

Post-mortem analysis of tensile samples was conducted using an optical microscope. Fracture surfaces, top, bottom, and side views of failed samples were studied to understand the crack propagation and failure patterns.

Representative microscope images are shown in Figure 11b,c. As we can see from Figure 11b, cracks happen in A30 and stop near the VMV reinforcement in one of the case two specimens. It is a result of the higher stiffness and strength of VMV than that of A30. The digital microscope images (Figure 11c) exhibit the uneven fracture surfaces of hybrid structures, which are captured for all other specimens as well. As revealed by the top view of the bottom half specimen (Figure 11c), small black lines and dots could be observed near the right extension. These are identified as the crack initiation points, which are caused by the stress concentration from the curved design of the Peano reinforcement. Then, the cracks propagate in the A30 matrix and form into a continuous crack, such as the long wave-shape black line shown in the top half of the specimen in Figure 11c. Additionally, no obvious delamination is captured at the A30/VMV interface, which indicates a reliable combining of the two different materials.

### 3.2. Compression Test Results and Discussions

**Figure 12:**
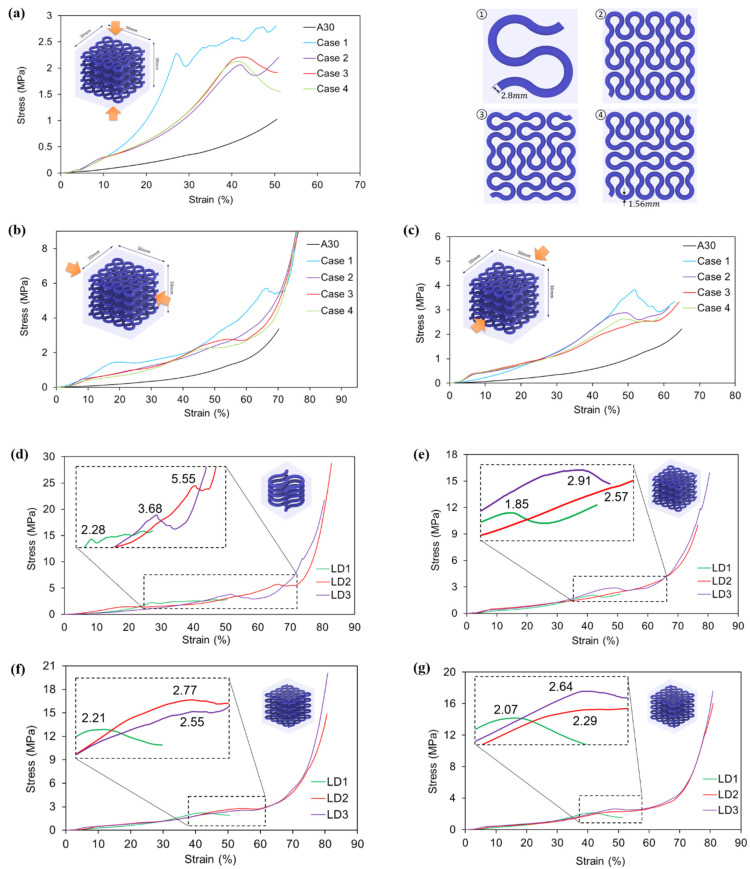

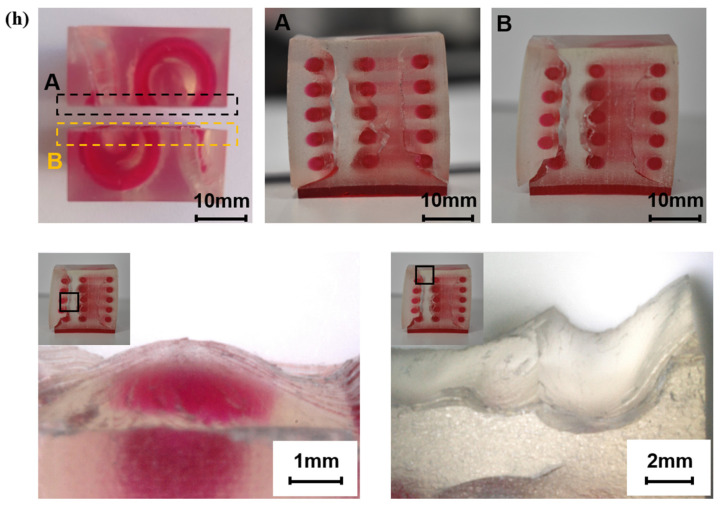
Stress-strain curves of different material designs, obtained from uniaxial compressive testings from (**a**) loading direction one (LD1); (**b**) loading direction two (LD2); (**c**) loading direction three (LD3). Comparisons of compressive stress-strain curves from three different loading directions for (d) design case one (pure horizontal/vertical first order); (**e**) design case two (pure horizontal/vertical second order); (**f**) design case three (mostly horizontal second order); (**g**) design case four (mostly vertical second order). (**h**) high resolution images (first row) and microscope images (bottom row) of case one, hybrid cube (pure horizontal/vertical first order) after compression from loading direction one. High resolution images show the cross-section A and B, from which cracks were found in the A30 matrix rather than A30/VMV interface. Microscope images show uneven fracture surfaces.

The results obtained from compressive tests are summarised in Figure 12, with comparisons made in three loading directions and among different hybrid materials.

Generally, the stress developed in all VMV reinforced hybrid samples is remarkably higher than that in homogenous A30 samples according to Figure 12a–c. The result indicates that the inclusion of the VMV Peano curve strengthens the A30 matrix regardless of loading directions. With regards to different reinforcement hierarchies, it could be observed that the composite materials with the first order reinforcement (case one) yield a higher compressive strength than the second order materials (case 2–4). This phenomenon ascribes to a larger diameter of reinforcement in the hybrid case one design (2.8 mm) than the other cases (1.56 mm), given the same volume fraction of 10% for all. It is also worth noticing that the responses of case two, case three, and case four are relatively similar for all three loading directions. It is caused by their similar amount of reinforcement at the cross-section perpendicular to the compression force.

As compressive cubes are designed anisotropic, compressive properties of four different hybrid cases are studied in different directions as shown in Figure 12d–g. The results elucidate that all hybrid cubes exhibit the lowest compressive strength subjected to loading direction one. Since Peano reinforcements lie in five-layers perpendicular to the compressive loading direction one, the amount of VMV material in the corresponding cross-section is the least among all three directions. For hybrid case one, the highest compressive strength (5.55 MPa) is captured in loading direction two and the second highest is found in loading direction three (3.68 MPa). This can be explained by the amount of reinforcement along the loading directions, which restrains the transverse expansion of A30 and thereby increases the strength. For the other three hybrid cases with second order reinforcement, there is only a slight difference between the compressive strength in loading direction two and loading direction three. As the hierarchy of reinforcement increases from first order to second order, the amount in the difference of reinforcement both lying along or perpendicular to loading direction two and loading direction three becomes very small.

To investigate the failure pattern of compression cubes, high resolution pictures and optical microscope images are taken to capture the fracture surfaces of failed samples. As compression samples are not broken into pieces, a bandsaw is used to cut the failed specimens in half along the compressive loading directions. Figure 12h shows the fracture surfaces of the hybrid case one specimen after compression from loading direction one. The high-resolution images of cross-section A and B, in the top row, clearly show the existence of wavy cracks in the A30 matrix. Supportive information is provided by the microscope images of the fracture surfaces (bottom row of Figure 12h). Transparent A30 is observed on top of VMV (bottom left image in Figure 12h), indicating the existence of a crack in the matrix rather than any debonding of A30/VMV. The results imply that the interface between two different materials is relatively strong. Furthermore, a concave surface is captured in A30 after the compression (bottom right image in Figure 12h). The reason behind this phenomenon is the same as the wave-shape cracks observed in tensile samples. To be more specific, it is caused by the stress concentration in the A30 matrix due to the curved design of VMV reinforcement.

## 4. Conclusions

In this paper, we designed novel composite materials inspired by the Peano curve. PolyJet 3D printing technology was used to fabricate samples with Agilus30 (A30) and VeroMagentaV (VMV). Mechanical properties were evaluated by mechanical tests, analytical predictions, and optical microscopy. Herein, the following conclusions are made:

- Compared to homogenous A30, all the hybrid tensile samples reinforced with VMV Peano curves yielded higher stiffness. This was attributed to the higher elastic modulus of VMV compared to A30. Consistent with the hypothesis, the elastic moduli obtained from tensile tests were within the range approximated from the rule of mixture (ROM) for composites.
- Hybrid tensile samples, which were designed with the second order Peano reinforcement, generally had a higher elastic modulus than tensile samples with the first order Peano reinforcement. It can be concluded that the second order reinforcement designs were more effective than the first order ones in terms of stiffness enhancement. For the hybrid tensile designs with the same reinforcement hierarchy, the pure horizontal alignment of reinforcement always provided a higher stiffness than the pure vertical designs owing to a higher ratio of reinforcement parallel to the tensile force.
- Regarding ultimate tensile strength, the improvement of hybrid designs compared to homogenous A30 was not obvious. Hierarchy and alignment of Peano reinforcements seemed to have little influence on the tensile strength as the stress could not be transferred effectively from matrix to reinforcement. However, an increasing trend of UTS could be witnessed with the growing ratio of reinforcements parallel to, and perpendicular to the loading direction. Experimental results were much lower than theoretical predictions due to the 3D manufacturing defects.
- The introduction of VMV Peano reinforcement in the A30 matrix resulted in higher stiffness and strength of the compression cubes. The first order reinforcement exhibited the best performance in all three directions among four different designs. The responses of three different second order designs were similar under compression.
- The second order compression cubes exhibited similar properties in loading direction two and loading direction three, due to the similar amount of reinforcement in all three cases along the compressive force.

## Figures and Tables

**Table 1:** Schematic diagrams and experimental pictures showing three different compressive loading directions (taking the case 2 design as a schematic example).

Loading Direction 1	Loading Direction 2	Loading Direction 3
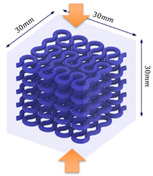	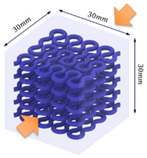	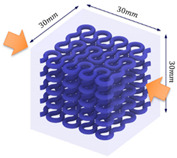
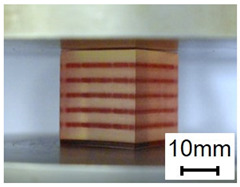	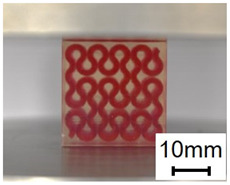	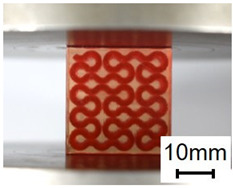

**Table 2:** Comparisons between experimental results and predictions using the ROM of average elastic modulus and ultimate tensile strength.

Material			Homogeneous		Composites					
			A30	VMV	Case 1	Case 2	Case 3	Case 4	Case 5	Case 6
Ratio of reinforcements parallel and perpendicular to the loading direction			-	-	6.00	0.40	2.35	0.34	1.21	0.68
Approximate volume fraction of reinforcement parallel to the loading direction			-	-	4.28%	1.43%	3.51%	1.27%	2.73%	2.02%
*E* (MPa)	Experiment		0.56~1.18	858	5.14	1.64	7.21	5.62	7.23	6.95
	ROM	Voigt’s (upper bound)	-	-	37.31~37.91	12.81~13.42	30.65~31.25	11.47~12.09	23.99~24.60	17.87~18.48
		Reuss’ (lower bound)	-	-	0.59~1.22	0.59~1.20	0.58~1.22	0.57~1.19	0.57~1.21	0.57~1.20
*UTS* (MPa)	Experiment		0.90	57.50	1.0340	0.79	0.91	0.89	0.93	0.94
	ROM		-	-	3.32	1.71	2.89	1.62	2.45	2.04
	Discrepancy		-	-	68.86%	53.57%	68.65%	45.31%	61.88%	54.12%

**Note**: The ROM denotes the general rule of mixture for composites; *E* denotes the elastic modulus measured from tensile testing; *UTS* denotes the average ultimate tensile strength measured from tensile testing.

## Data Availability

Not applicable.
